# Understanding the body image perception of pregnant women during their third trimester in a tertiary care setting in Southern India

**DOI:** 10.1186/s12884-024-06864-7

**Published:** 2024-10-14

**Authors:** Ketaki Desai, Deepalaxmi Paresh Poojari, T.S. Shwetha, Rajani Upadhyaya, Preetha Ramachandra

**Affiliations:** 1 Department of Physiotherapy, Manipal College of Health Professions, Manipal Academy of Higher Education, Manipal, Karnataka India; 2 Department of Clinical Psychology, Manipal College of Health Professions, Manipal Academy of Higher Education, Manipal, Karnataka India; 3 Department of Obstetrics and Gynecology, Kasturba Medical College, Manipal, Manipal Academy of Higher Education, Manipal, Karnataka India

**Keywords:** Body image, Perception, Pregnancy, Third trimester

## Abstract

**Background:**

Pregnancy is a known physiological phenomenon characterized by various changes in the body. The physical and physiological changes that occur during pregnancy may impact the body image which may lead to implications such as body image issues or poor eating habits among pregnant women. This study aimed to analyze the body image perception of pregnant women in their third trimester.

**Methods:**

This cross-sectional survey which involved the administration of a Multidimensional Body Self-Relations Questionnaire (MBSRQ), was conducted among pregnant women between 28 and 40 weeks of gestation in a tertiary care setting in Southern India. Descriptive statistics were used to report the demographic characteristics of the respondents. A one-sample t-test was used to analyze the difference between the present sample scores and the published norms of MBSRQ. Univariate Logistic Regression was done to find the association between the demographic variables and subdomains of MBSRQ.

**Results:**

The mean age of the respondents (*n* = 246) was 29.5 years, and the period of gestation was 33.4 weeks. With mean scores of subscales of MSRQ as reference values, a greater proportion of women had higher scores on appearance orientation (52.44%), health evaluation (56.91%), and illness orientation (55.28%). Respondents scored less on appearance evaluation (52.03%) and body areas satisfaction scale (50.41%). The study found that pre-pregnancy BMI, abdominal circumference, and weight gain during pregnancy were associated with appearance orientation, overweight preoccupation, and self-classified weight. Health evaluation was associated with weight gain and Instagram use, while moderate-intensity physical activity during pregnancy was associated with higher health orientation.

**Conclusion:**

Although pregnant women in our setting during the third trimester were oriented towards their appearance and considered themselves healthy and fit, almost half of the respondents reported dissatisfaction with their changing bodies and appearance. Self-reported physical activity status, body mass index, weight gain, level of education, use of Instagram app, and type of family were factors found to affect pregnant body image perception. Hence, we conclude that body image perception is affected during pregnancy, and healthcare professionals should be aware of this, and the factors associated with it while addressing the health of pregnant women.

**Clinical trial registration details:**

The study was registered under the Clinical Trials Registry- India: CTRI/2023/08/056524. https://ctri.nic.in/Clinicaltrials/rmaindet.php?trialid=89771&EncHid=39880.12369&modid=1&compid=19.

**Supplementary Information:**

The online version contains supplementary material available at 10.1186/s12884-024-06864-7.

## Background

Pregnancy is a known physiological phenomenon characterised by changes in various body systems such as the musculoskeletal, metabolic, cardiovascular, hormonal and endocrine systems [1]. These changes are internal, but they have external manifestations such as changes in the skin, size, and shape of body parts such as the abdomen, breasts, hips, buttocks and lower limbs, and posture causing changes in the body including functional changes in strength and endurance. Weight gain is essential for a healthy pregnancy as it helps in fetal growth and fulfils increasing maternal demands. The third trimester is particularly a period of observable external changes in pregnancy [2]. The literature surrounding body image describes it as a construct with cognitive, perceptive, affective, and behavioural dimensions [3]. Previous qualitative studies have reported that social and emotional support received by pregnant women including partners’ support, can immensely affect their body perception, directing it towards a positive body image. However, lack of social support, comments about the growing abdomen and weight gain, changes in the size of clothing, and lack of assistance from the consulting doctors were observed to be some factors causing negative body perception [4–6].

The use of social media by pregnant women is a known influencing factor in the way that body-positive content leads to more satisfaction, while idealized pictures of the pregnant body may cause downward self-comparison and negative affect [7]. Apart from perceived social and emotional support from friends, family, and partners and media influence, a better body image has also been associated with older age, multiparity, better socioeconomic status, lower pre-pregnancy body mass index, gestational weight gain up to mid-pregnancy, and pre-pregnancy body image perception. However, most of the studies are qualitative, and the data from cross-sectional and prospective remains inconclusive about the factors demanding more studies [3].

The development of body image perception during pregnancy may be distinct. A Western study surrounding the experiences of women of their pregnant bodies has reported that women feel excused from abiding by the ‘perfect body’ standards during pregnancy [4]. However, there is a lack of understanding about how women with Indian backgrounds perceive the changes in their body during pregnancy. The Multidimensional Body Self-Relations Questionnaire is one of the tools for evaluating all aspects of body perception [8]. This scale has 10 subscales that extensively explore body image perception, and the use of this scale in pregnant women has the potential to provide extensive information about the body image issues in this population which will contribute to the knowledge of body image among Indian pregnant women to the existing literature [6, 9, 10]. Therefore, the present study aims to analyze the body image perception of pregnant women in their third trimester.

## Methods

### Research design and participants

This cross-sectional study was conducted in a university tertiary care hospital in Southern India. Pregnant women who visited the outpatient Department of Obstetrics and Gynecology for antenatal scans between August 2023 to February 2024 were screened by the principal investigator for inclusion and exclusion criteria. The eligible women were explained about the study and were recruited by the principal investigator if they voluntarily consented to respond to the questionnaire. Pregnant women who were aged between 18 and 45 years, in their third trimester (28 to 40 weeks of gestation), and could read and write either English or Kannada were included in the study. Women who conceived through artificial reproductive treatment, a high-risk pregnancy (women with short cervix who were advised bed rest, multifetal pregnancy) or who had musculoskeletal, neurological or cardiac conditions affecting their physical functioning were excluded from the study. We calculated the sample size as 246 using the estimation of proportion formula with a proportion in population of 20%. ($\:Sample\:size=\frac{{Z}_{{(1-\frac{\alpha\:}{2})}^{2\:}}\times\:p(1-p)\:}{{d}^{2}}\:\:$ where $\:{Z}_{(1-\frac{\alpha\:}{2})}$ is 1.96 at 5% error, p is the proportion of 0.2 and d is the precision which is 0.05) [11].

### Tool

The Multidimensional Body Self-Relations Questionnaire (MBSRQ) was used to assess the body image perception of pregnant women. The MBSRQ is a 69-item self-report tool to evaluate body image from a multidimensional perspective. It includes seven subscales namely, appearance evaluation, appearance orientation, health evaluation, health orientation, fitness evaluation, fitness orientation, and illness orientation and three additional subscales viz. body areas satisfaction scale, overweight preoccupation and self-classified weight. All the subscales have acceptable internal consistencies [12, 13]. Higher scores indicate better self-perception, and lower scores indicate poorer self-perception in the domain except in overweight preoccupation and self-classified weight subscales which have higher scores signifying poor perception and lower scores denoting better perception about one’s weight [13].

### Research procedure

We obtained written informed consent from all the participating women. The women were given the English or Kannada version of MBSRQ as per their preference. Any participant queries about the questionnaire were addressed by the principal investigator. Data regarding sociodemographic details (age, pre-pregnancy, and current weight, height, geographic location, highest level of education and occupation), self-reported variables like physical activity status (self-perceived level of physical activity), sleep quality (one’s rating of their quality of sleep), social media usage (type of social media apps used and the duration of usage in a day), partner satisfaction (one’s rating for their overall satisfaction with their partner) and family type (nuclear family which includes two to five members or joint family which includes more than five members where two or more generations of the same paternal or maternal line live together) were recorded. Variables such as sleep quality and partner satisfaction were classified based on the rating given by the women for these on a 10-point rating scale [14].

The abdominal circumference was measured using an inch tape at the umbilical level. Body Mass Index (BMI) was calculated by the principal investigator to calculate the pre-pregnancy BMI and current BMI at the time of data collection. Body weight was recorded using a weighing scale and the height was measured using a stadiometer. The WHO criterion for BMI classification was used to classify them as underweight, normal weight, overweight or obese [15]. Gestational Weight Gain (GWG) was calculated by subtracting the body weight (self-reported) at the time of conception from the weight at the point of gestation during participant recruitment. Based on the GWG, the women were classified into the categories of Institute of Medicine Guidelines for GWG 2009 as less than the recommendation, within normal range, and above recommendation [16].

### Data analysis

Data was analysed using Jamovi 2.4.14 software. Descriptive statistics was used to report the demographic characteristics. Shapiro-Wilk Test was done to test the normality of all the variables included. Mean and standard deviation were calculated for the continuous variables and the scores of MBSRQ. A one-sample t-test was used to analyze the difference between the mean scores of the present sample and the published norms of MBSRQ for women [12, 13]. Univariate Logistic Regression was done to find the association between the variables. The association between sociodemographic and self-reported personal characteristics of the respondents as the independent variables was analysed with each MBSRQ subscale as the dependent variable. The association was considered statistically significant when *p* < 0.05.

## Results

**Fig. 1:**
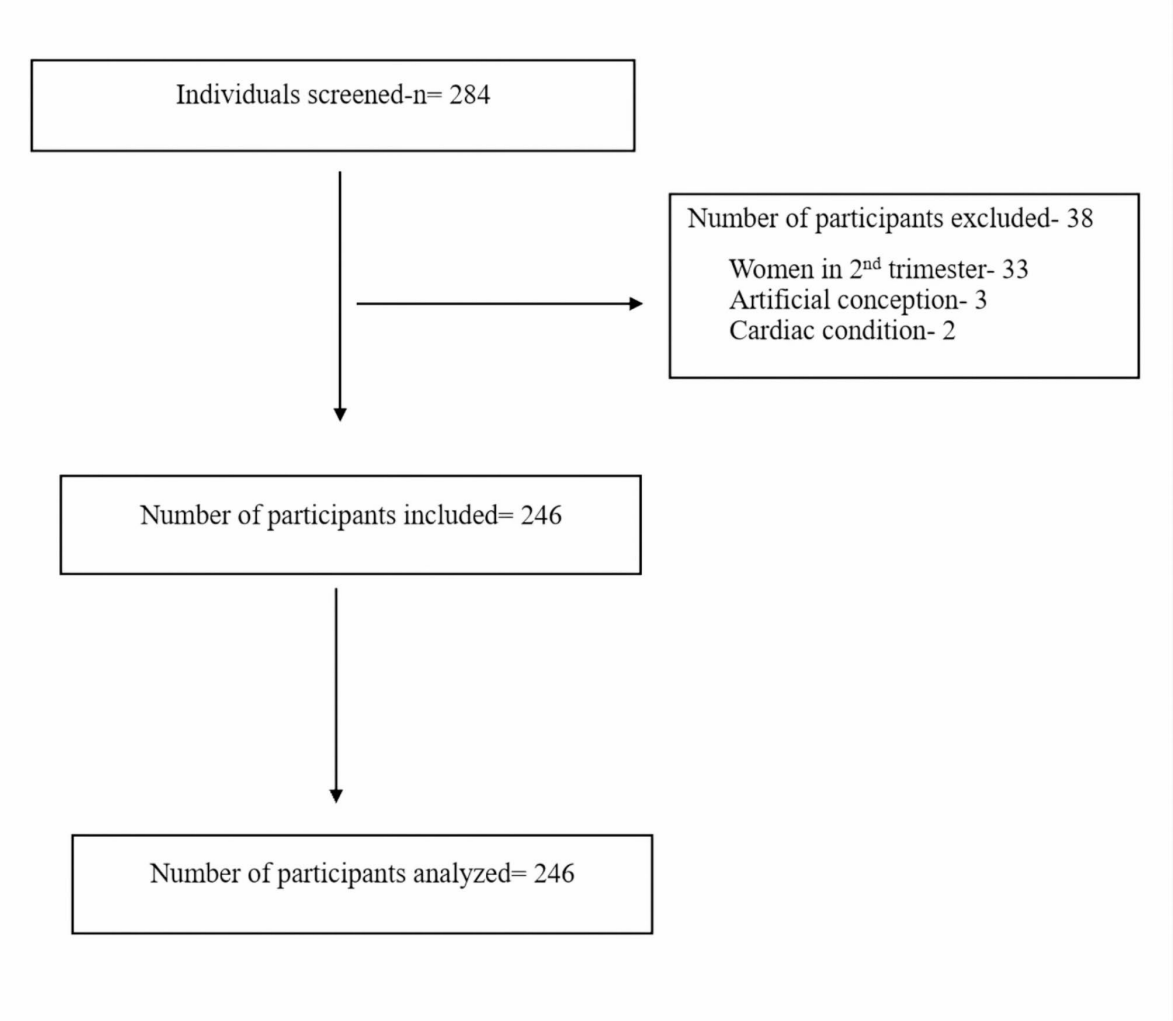
Flow of participants in the study

The flow of recruitment of participants has been depicted in Fig. 1.

The mean age of the participants was 29.5 years, and the period of gestation was 33.4 weeks. The sociodemographic characteristics of the participants are shown in Table 1. All participants were educated and about half of them were not in paid employment. The gestational weight gain of many women was less than the recommendations for their weight category as per IOM guidelines, 2009 which provide an ideal range for pregnancy weight gain for women based on their pre-pregnancy BMI [16].

**Table 1:** Participant characteristics

Variables		*N* (%)
**Geographic Location**		
• *Rural* • *Urban*		152 (61.79) 94 (38.21)
**Highest level of Education**		
• *High School* • *Senior Secondary* • *Graduate* • *Postgraduate*		15 (6.09) 30 (12.19) 142 (57.72) 59 (23.98)
**Occupation**		
• *Not in paid employment* • *Desk workers* • *Healthcare Professionals* • *Teachers* • *Others*		132 (53.66) 62 (25.20) 25 (10.16) 12 (4.87) 15 (6.09)
**BMI (kg/** $\:{\varvec{m}}^{2}$ **)**		
*BMI at 3rd trimester*		
• *Underweight* • *Normal* • *Overweight* • *Obese*		3 (1.22) 93 (37.80) 111 (45. 12) 39 (15.86)
*Self-reported weight at conception*		
• *Underweight* • *Normal* • *Overweight* • *Obese*		29 (11.79) 164 (65.67) 40 (16.26) 13 (5.29)
**Weight Gain (kg)***(As per IOM guidelines*,* 2009)*		
• *Less than recommended norms* • *Within recommended norms* • *More than recommended norms*		148 (60.16) 63 (25.61) 35 (14.23)

*IOM*- Institute of Medicine

*As per IOM Guidelines*,* recommended range of weight gain for women who were underweight*,* normal weight*,* overweight*,* and obese before pregnancy is 12.5–18 kg*,* 11.5–16 kg*,* 7–11.5 kg*,* and 5–9 kg respectively*

The self-reported variables about their pre-pregnancy and present physical activity status, types of exercise performed, sleep quality, use of social media, types of apps used, partner satisfaction, and type of family are depicted in Table 2. As per the reported status, 70.73% of women were inactive before pregnancy which reduced to 43.50% during their third trimester along with an increase in the percentage of women reporting light physical activity during pregnancy. Walking was the major form of exercise performed by the participants during pregnancy. The other variables suggest that most of the pregnant women rated their sleep quality as good and used social media. Most of them were very satisfied with their partners and belonged to nuclear families (lived with partner and child(ren).

**Table 2:** Self-reported activity and personal characteristics

Variables	*N* (%)
**Self-reported physical activity status**	
*Pre-pregnancy*	
• *Inactive* • *Light* • *Moderate*	174 (70.73) 45 (18.29) 27 (10.98)
*Present*	
• *Inactive* • *Light* • *Moderate*	107 (43.50) 102 (41.46) 37 (15.04)
**Type of exercise**	
• Walking • Yoga • Antenatal Exercise • Others	117 (47.56) 11 (4.47) 16 (6.50) 13 (5.28)
**Self-reported sleep quality**	
• *Average* • *Good* • *Very Good*	45 (18.29) 164 (66.67) 37 (15.04)
**Use of social media**	
• *Present* • *Absent*	180 (73.17) 66 (26.83)
**Apps used**	
• *Instagram* • *Facebook* • *Snapchat* • *Others*	143 (41.87) 113 (45.93) 19 (7.72) 10 (4.07)
**Partner Satisfaction**	
• *Unsatisfied* • *Satisfied* • *Very satisfied*	3 (1.22) 32 (12.98) 211 (85.77)
**Family Type**	
• *Joint* • *Nuclear*	58 (23.58) 188 (76.42)

The mean scores of our respondents were used for categorizing the individuals into high scores and low scores on the subscales of MBSRQ. A significant difference was noted between the present sample mean scores compared to the published norms of MBSRQ [13, 17] in all the subscales as represented in Table 3. We observed that in our population, the mean values were higher for Appearance Orientation, Health orientation, Fitness evaluation, Fitness Orientation, Illness orientation, Body area satisfaction and Overweight pre-occupation subscales compared to the published norms.

**Table 3:** Comparison of MBSRQ mean scores and standard deviation of present sample to published norms

MBSRQ Subscale	Present Sample	Published Norms	*p*-value
	Mean (SD)	Mean (SD)	
Appearance evaluation	3.59 (0.50)	3.36 (0.87)	< 0.001
Appearance Orientation	3.26 (0.52)	3.91 (0.60)	< 0.001
Health Evaluation	3.46 (0.52)	3.86 (0.80)	< 0.001
Health Orientation	3.94 (0.51)	3.75 (0.70)	< 0.001
Fitness Evaluation	3.58 (0.72)	3.48 (0.97)	0.002
Fitness Orientation	3.49 (0.46)	3.20 (0.85)	< 0.001
Illness Orientation	3.90 (0.71)	3.21 (0.84)	< 0.001
Body Areas Satisfaction Scale	3.90 (0.69)	3.23 (0.74)	< 0.001
Overweight Preoccupation	2.41 (0.74)	0.03 (0.96)	< 0.001
Self-classified Weight	3.10 (0.62)	3.57 (0.73)	< 0.001

**Fig. 2:**
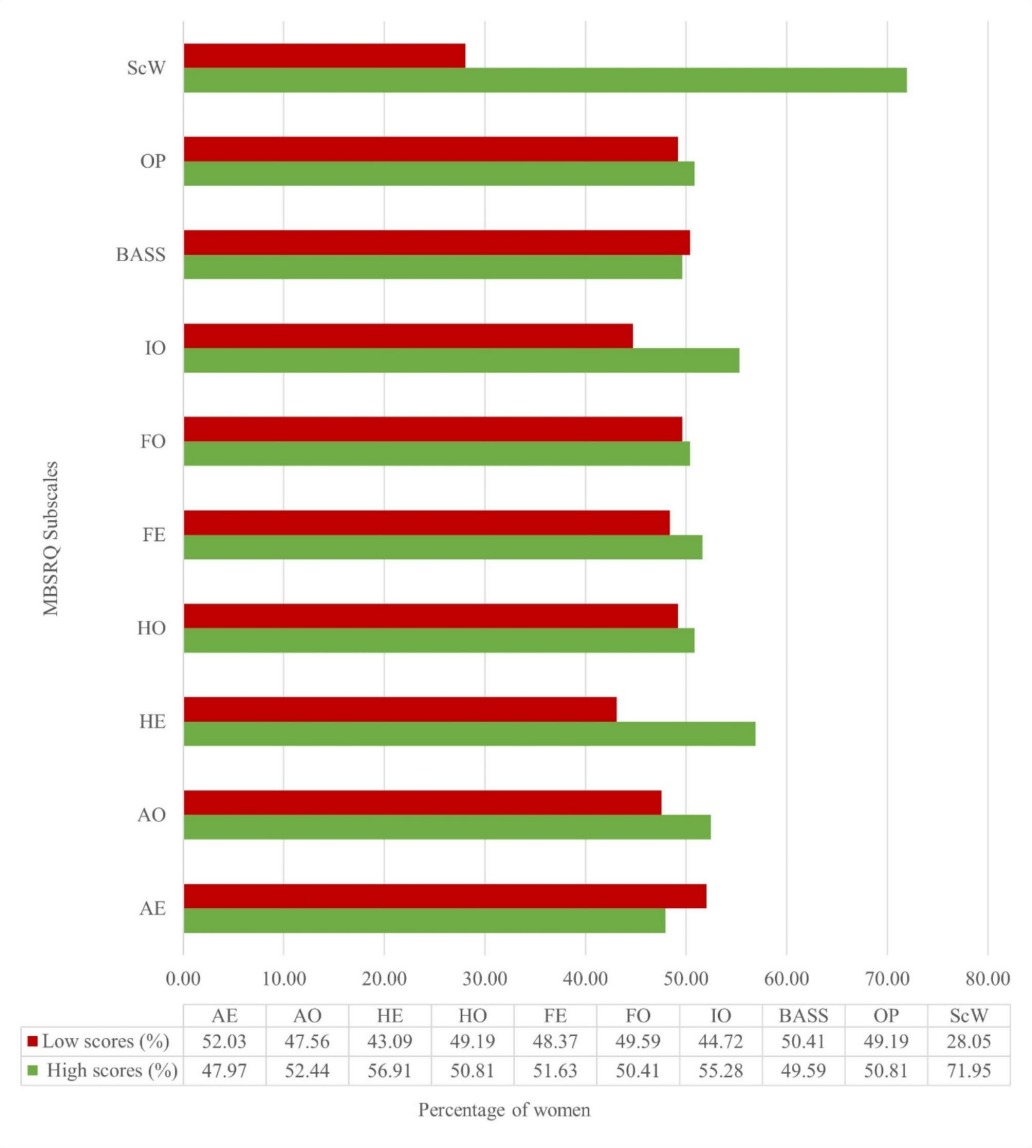
Subscales of MBSRQ represented as frequencies (*n* = 246) *AE-*Appearance Evaluation, *AO-*Appearance Orientation, *HE-*Health Evaluation, *HO-*Health Orientation, *FE-*Fitness Evaluation, *FO-*Fitness Orientation, *IO-*Illness Orientation, *BASS-*Body Areas Satisfaction Scale, *OP-*Overweight Preoccupation, *ScW-*Self-classified Weight

The scores of the participants on the subscales of MBSRQ are depicted in Fig. 2 as percentages of women with high and low scores. We found that a greater percentage of pregnant women scored more than the mean values in the subscales- Self-classified Weight, Illness Orientation, Health Evaluation, and Appearance Orientation. The subscale where a greater percentage of pregnant women scored less than the mean values was Appearance Evaluation.

Table 4 indicates the results of Univariate logistic regression analysis of factors that were found to be significantly associated with each subscale of MBSRQ. We found that the pre-pregnancy BMI, BMI at 3rd trimester and abdominal circumference were the factors which were associated with Appearance Orientation, Overweight Preoccupation and Self-Classified Weight. Health Evaluation was observed to be associated with weight gained during pregnancy and the use of Instagram while those who reported their pre-pregnancy physical activity status as moderate intensity compared to inactivity had higher health orientation in third trimester. Fitness Evaluation was linked to the employment status with poor evaluation among employed women and the type of family was associated with Fitness Orientation. Moderate intensity physical activity status before pregnancy was associated with both Fitness Evaluation and Orientation. We identified education level and the type of family to be influencing factors for Illness Orientation. Good sleep quality compared to average rating was associated with greater body areas satisfaction. In addition to those mentioned above, the factors associated with Self-Classified Weight were weight gain, use of Instagram and pre-pregnancy physical activity status of moderate intensity compared to inactive.

**Table 4:** Results of Univariate logistic regression analysis of factors related to MBSRQ Subscales

Variables	AO		HE		HO			FE		FO			IO		BASS			OP			ScW	
	OR (95% CI)	p	OR (95% CI)	p	OR (95% CI)	p	OR (95% CI)		p	OR (95% CI)	p	OR (95% CI)		p	OR (95%CI)	p	OR (95%CI)		p	OR (95%CI)		p
Age	-	-	-	-	-	-	-		-	-	-	-		-	-	-	-		-	-		-
Education Level																						
Graduate- Non-graduate	-	-	-	-	-	-	-		-	-	-	2.13 (1.10- 4.12)		0.02	-	-	-		-	-		-
Occupation																						
Employed- Homemakers	-	-	-	-	-	-	0.46 (0.27–0.76)		0.003	-	-	-		-	-	-	-		-	-		-
Pre-pregnancy BMI	0.90 (0.85–0.97)	0.004	-	-	-	-	-		-	-	-	-		-	-	-	0.91 (0.85–0.97)		0.004	0.88 (0.82–0.94)		< 0.001
BMI at 3rd trimester	0.91 (0.86–0.98)	0.01	-	-	-	-	-		-	-	-	-		-	-	-	0.90 (0.84–0.97)		0.003	0.80 (0.74–0.87)		< 0.001
Abdominal Circumference	0.95 (0.93–0.98)	0.008	-	-	-	-	-		-	-	-	-		-	-	-	0.96 (0.93–0.99)		0.004	0.93 (0.91–0.97)		< 0.001
Weight Gain																						
• Less than norms • More than norms	- -	- -	0.52 (0.27–0.97) 0.32 (0.13–0.76)	0.04 0.01	- -	- -	- -		- -	- -	- -	- -		- -	- -	- -	- -		- -	- -		- -
Weight Gain (kgs)	-	-	-	-	-	-	-		-	-	-	-		-	-	-	-		-	0.93 (0.88–0.98)		0.005
Use of Social Media Apps																						
• Instagram • Facebook	- -	- -	0.56 (0.33–0.94) -	0.02 -	- -	- -	- -		- -	- -	- -	- -		- -	- -	- -	- -		- -	0.55 (0.31–0.99) - -		0.049 -
Previous self-reported PA status																						
• Moderate- Inactive • Moderate- Light	- -	- -	- -	- -	2.54 (1.05–6.12) -	0.04 -	3.21 (1.29–7.98) -		0.01 -	5.41 (1.96–14.95) 3.85 (1.23–11.96)	0.001 0.02	- -		- -	- -	- -	- -		- -	0.26(0.11–0.60) -		0.002 -
Family Type																						
Nuclear- Joint	-	-	-	-	-	-	-		-	0.54 (0.29–0.98)	0.04	0.34 (0.17- 0.65)		0.001	-	-	-		-	-		-
Self-reported sleep quality																						
• Good- Average • Very Good- Average	- -	- -	- -	- -	- -	- -	- -		- -	- -	- -	- -		- -	2.05 (1.03–4.05) -	0.04 -	- -		- -	- -		- -

*AE-*Appearance Evaluation, *AO-*Appearance Orientation, *HE-*Health Evaluation, *HO-*Health Orientation, *FE-*Fitness Evaluation, *FO-*Fitness Orientation, *IO-*Illness Orientation, *BASS-*Body Areas Satisfaction Scale, *OP-*Overweight Preoccupation, *ScW-*Self-classified Weight, *BMI*- Body Mass Index, *OR*- Odd’s Ratio, *CI*- Confidence Interval

Additional factors that did not show an association between factors and MBSRQ subscales are detailed in Supplementary File 1.

## Discussion

The study intended to understand the body image of pregnant women in the third trimester. We found that pregnant women had body image issues in their third trimester of pregnancy. Although half of the sample perceived themselves as attractive and fit and invested efforts that they considered would maintain their health, fitness, and appearance, an equal percentage of pregnant women also showed dissatisfaction with changing body dimensions. Self-classified weight indicated that most of the women considered themselves as normal weight or underweight and were also preoccupied with being overweight.

### Appearance evaluation, appearance orientation and body areas satisfaction

We identified that half of our sample assessed their appearance negatively, indicating that they were dissatisfied with the way they looked. Previous study conducted on the Western population have reported that the changes in the skin, shape and size of the body and sexual attractiveness were found to be perceived negatively and these were influenced by the opinions of their family, partner, and friends [2]. In a previous study, it was reported that age was a predictor of body image self-evaluation in mid-pregnancy, and it became insignificant at the end of pregnancy [3]. It was also reported that the time point of pregnancy at which the prediction is being made affects the association. Our study results also showed that age did not show an association with the subscales of MBSRQ. In the third trimester, pregnant women may be more concerned about their baby and delivery outcomes than the appearance of their body. In the present study, half of the sample were invested in grooming behaviour and maintaining an appearance in which they think they look good. A previous qualitative study has reported that pregnant women felt satisfied with their bodies till they were able to wear their pre-pregnancy clothing, but as the clothes began to not fit, they were aware of their weight gain and perceived dissatisfaction with their bodies [2]. We can infer from our findings that, women with lower pre-pregnancy BMI may be more invested in their appearance with an increase in their weight and abdominal size.

Our study results also suggest that women who rated their sleep as good were more satisfied with their body parts compared to poor sleep quality. Poor sleep quality has been stated to be a risk factor for poor satisfaction with strength, appearance, and sexual attractiveness [3]. In our study, we had an almost equal proportion of respondents who scored high as well as low on body area satisfaction. Previous study has reported mixed opinion among women regarding satisfaction with body areas depending on which part gains more “fat”. The increasing size of breasts, widening hips and waist, and skin changes over the face and abdomen have been reported to cause more dissatisfaction than the growing abdomen [2]. Pre-pregnancy body shape and size, and self-perceived ideal body for women are known to affect the internalization of own body image during pregnancy [18].

### Health evaluation and orientation

More than half of the sample in the current study evaluated themselves to be healthy. Those who had normal weight gain had better health evaluations compared to the women who gained weight above or below the norms. As per this categorization, weight gain and growing belly may be used as a measure of fetal well-being and healthy status of the mother and child compared to less or excess weight gain [4]. Compared to non-users of social media apps like Instagram and Facebook, the users had poorer scores for health evaluation. A well-established effect of social media exists on the body image of pregnant women as most of them access it for health and pregnancy-related information [19]. Social media platforms have a vast range of content which includes images of pregnant high-profile celebrities and social media influencers who promote diet and exercise habits for pregnancy. Women generally have positive attitudes towards influencers, believing them credible sources of information [20]. Social media content rumination and time spent on thinking can influence positive or negative body attitudes among pregnant women [21]. However, these studies were from a culturally distinct Western population and none of them included South Asian women. The source for most of the health-related practices during pregnancy in most of the South Asian nations is the elder women in the family [22]. Hence, whether the poor or better evaluation of health in our women in the third trimester arises due to social media use is unclear as we did not investigate the type of social media content that our sample accessed.

Half of our respondents appeared to have a better health orientation and were conscious about their physical health. They followed healthy lifestyle practices such as engaging in regular exercise and eating food with health benefits. However, an equivalent proportion of women having lower health orientation can be attributed that women did not take conscious efforts to enhance their health during pregnancy. Pregnancy-related lifestyle habits have been linked to favourable outcomes [23, 24].

### Fitness evaluation and orientation

An even distribution is evident in the scores of fitness evaluation and orientation. We observed that occupation was found to be a significant factor for fitness evaluation where employed women had poor scores compared to homemakers. Most of our employed women were desk job workers and hence had prolonged hours of working in a stationary position contrary to homemakers who performed varied household activities throughout the day which may impact the perception of their fitness levels. Our results are in line with previous literature which has reported similar findings [24]. Fitness orientation reflected an association with pre-pregnancy moderate-intensity exercise indicating that previously active individuals had more fitness inclination even during late pregnancy compared to women who were inactive or had low physical activity levels before pregnancy. According to previous studies, women who performed high levels of exercise (90 min per week of moderate-intensity exercise) before pregnancy had greater body satisfaction in late pregnancy compared to women who did not exercise or performed low-intensity exercises [3, 25]. We have observed a shift of inactive women before conception to start walking during the third trimester. Literature has stated that conventional practice encouraged pregnant women to walk and perform all their daily activities during pregnancy for beneficial labour outcomes [26, 27]. Women from nuclear families had lower fitness orientation compared to joint families. Factors such as household responsibilities, multiparity, motivation to exercise and family beliefs about exercise during pregnancy might play a role among others [28, 29].

### Illness orientation

The findings of illness orientation imply that women who had an education level of graduation or above were more inclined towards seeking attention for their health if any signs of illness were observed. This inference is in line with previous studies which report that women who had better education were more aware of signs of illness and had a birth plan to follow. It is reported that women must be educated about warning signs of any danger which improves their understanding of seeking medical help [30, 31]. The reason for more participants having higher illness orientation could be that our population belonged to the third trimester and all of them had frequent antenatal check-ups as per routine protocol during this period, with signs of danger being explained to them by the clinicians.

### Self-classified weight and overweight preoccupation

Most women in the present study are seen to have normal BMI before pregnancy and a minimal number of women have gained weight within the recommended norms [16]. The women with higher BMI before pregnancy or during the third trimester, and those with greater abdominal circumference had a higher preoccupation with their weight. Women with higher pre-pregnancy BMI also self-classified themselves as overweight in late pregnancy on self-classified weight compared to those with low or normal BMI. A study among Japanese pregnant women in their second trimester reported that their body dissatisfaction increased with increasing BMI, and they tried to control their weight gain even during pregnancy irrespective of their pre-pregnancy BMI. They also overestimated their body size during pregnancy [32]. However, in our population, gestational weight gain was not found to be significantly associated with overweight preoccupation. Nevertheless, women who had higher pre-pregnancy BMI continued to label themselves overweight irrespective of gain in weight. Pre-pregnancy body image may have played a major role in this perception which was also suggested earlier [18].

### Strengths and limitations

We acknowledge a few limitations of our study. A few factors such as parity and the planning of pregnancy were not taken into consideration in the study. As few variables like pre-pregnancy BMI and pre-pregnancy activity level are self-reported, and hence, there is a chance of recall bias. Since our respondent’s first-trimester visits to the hospital varied, we collected self-reported weight as the body weight at the time of conception which may influence the actual values of gestational weight gain. The use of a questionnaire to assess body image limits the identification of possible aspects other than those mentioned in the questionnaire which can be a part of the women’s perception. The present study was conducted in a single tertiary care setting of a middle-income country. This limits its generalizability to a wider population. We cannot identify causal relationships since it is a cross-sectional study. A prospective study designed to gauge body image perception across various time points extending from pre-pregnancy, through pregnancy and postpartum may provide a clearer understanding of the changes in body image perception.

## Conclusion

Although pregnant women in our setting during the third trimester were oriented towards maintaining an attractive appearance and viewed themselves as healthy and fit, almost half of the respondents reported dissatisfaction with their changing bodies and appearance. They were prompt in seeking medical help in case of signs of illness. Self-reported physical activity status, body mass index, weight gain, level of education, use of Instagram app, and type of family were factors found to affect pregnant body image perception. Hence, we conclude that body image perception is affected during pregnancy, and healthcare professionals should be aware of this and the factors affecting body image. Based on our study results, interventions must include psychological components that address body image issues in addition to the emphasis given to physical health.

## Electronic supplementary material

Below is the link to the electronic supplementary material.

Supplementary Material 1

## Data Availability

The datasets used and/or analysed during the current study are available from the corresponding author on reasonable request.

## Abbreviations

BMI: Body Mass Index
MBSRQ: Multidimensional Body Self-Relations Questionnaire
