# Supplementary material for: Understanding the body image perception of pregnant women during their third trimester in a tertiary care setting in Southern India
Source: BMC Pregnancy Childbirth. 2024 Oct 14;24:671. doi: 10.1186/s12884-024-06864-7 (PMC11476645; doi:10.1186/s12884-024-06864-7)
Supplement: Supplementary file 1 — Supplementary Material 1 [file 12884_2024_6864_MOESM1_ESM.pdf]

Supplementary File 1: Results of Univariate Logistic Regression Analysis of Factors not associated with MBSRQ subscales:

	**AE**		**AO**		**HE**		**HO**		**FE**		**FO**		**IO**		**BASS**		**OP**		**ScW**	
Variables	OR (95% CI)	p	OR (95% CI)	p	OR (95% CI)	p	OR (95% CI)	p	OR (95% CI)	p	OR (95% CI)	p	OR (95% CI)	p	OR (95%CI)	p	OR (95%CI)	p	OR (95%CI)	p
Age	0.98(0.91-1.04)	0.45	0.97(0.91-1.04)	0.45	1.02(0.95-1.10)	0.53	1.02(0.95-1.10)	0.50	1.03(0.96-1.10)	0.41	0.99(0.93-1.07)	0.98	1.01(0.94-1.08)	0.77	1.04 (0.97-1.11)	0.31	1.04(0.67-1.11)	0.30	1.01(0.93-1.09)	0.78
Education Level<br>Graduate- Non-graduate	1.07(0.56-2.04)	0.85	0.95(0.50-1.82)	0.89	0.60(0.30-1.19)	0.15	1.53(0.79-2.93)	0.20	1.03(0.54-1.96)	0.94	1.34(0.70-2.57)	0.38	-	-	1.44(0.75-2.766)	0.27	0.99(0.52-1.88)	0.96	0.92(0.44-1.90)	0.82
Occupation<br>Employed- Homemakers	0.90(0.54-1.48)	0.67	0.73(0.44-1.22)	0.23	0.82(0.50-1.37)	0.45	0.83(0.50-1.36)	0.45	-	-	0.75(0.45-1.23)	0.25	0.82(0.94-1.36)	0.44	0.90(0.55-1.49)	0.69	1.39(0.84-2.31)	0.19	0.85(0.49-1.48)	0.56
Location<br>Urban- Rural	0.80(0.48-1.35)	0.41	0.75(0.44-1.26)	0.28	1.28(0.75-2.16)	0.35	0.72(0.43-1.21)	0.21	1.19(0.70-1.99)	0.52	0.69(0.41-1.16)	0.16	1.33(0.78-2.23)	0.29	0.78(0.46-1.30)	0.34	1.02(0.61-1.70)	0.95	1.34(0.74-2.40)	0.33
Pre-pregnancy BMI	0.98(0.92-1.05)	0.63	-	-	0.98(0.92-1.04)	0.45	0.94(0.89-1.01)	0.08	0.99(0.93-1.05)	0.69	0.98(0.92-1.04)	0.53	0.97(0.91-1.03)	0.36	0.99(0.93-1.06)	0.87	-	-	-	-
BMI at 3rd trimester	1.00(0.94-1.07)	0.89	-	-	0.97(0.91-1.04)	0.42	0.96(0.90-1.02)	0.16	1.00(0.94-1.07)	0.85	0.99(0.93-1.05)	0.71	1.00(0.94-1.07)	0.88	0.97(0.91-1.03)	0.32	-	-	-	-
Abdominal Circumference	1.00(0.97-1.03)	0.78	0.95 (0.93-0.98)	0.008	0.97(0.94-1.00)	0.052	0.97(0.94-1.00)	0.07	1.00(0.97-1.03)	0.80	0.99(0.96-1.02)	0.41	0.99(0.96-1.02)	0.73	0.97(0.95-1.00)	0.08	-	-	-	-
Weight Gain (kgs)	1.02(0.97-1.06)	0.39	1.01(0.97-1.06)	0.53	0.99(0.95-1.04)	0.92	1.01(0.97-1.05)	0.57	1.02(0.98-1.07)	0.21	1.01(0.97-1.06)	0.49	1.04(0.99-1.09)	0.07	0.97(0.93-1.01)	0.14	0.98(0.95-1.04)	0.88	-	-
Use of Social Media Apps																				
• Instagram	1.17(0.70-1.95)	0.53	0.73(0.44-1.22)	0.23	-	-	0.89(0.54-1.49)	0.67	0.95(0.57-1.57)	0.83	1.06(0.64-1.76)	0.81	0.66(0.39-1.11)	0.12	1.00(0.60-1.67)	0.98	1.02(0.62-1.70)	0.93	-	-
• Facebook	0.86(0.52-1.43)	0.57	0.92(0.55-1.53)	0.76	0.65 (0.39-1.09)	0.10	0.70(0.42-1.16)	0.17	1.19(0.72-1.97)	0.49	0.82(0.49-1.36)	0.45	0.61(0.37-1.01)	0.055	0.87(0.53-1.45)	0.60	0.79(0.48-1.32)	0.38	0.76(0.44-1.34)	0.35

Previous self-reported PA status																				
• Moderate-Inactive			1.31(0.68-2.53)	0.41	0.59(0.31-1.15)	0.12	-	-	-	-	-	-	1.45(0.63-3.34)	0.39	1.67(0.73-3.80)	0.22	0.73(0.32-1.65)	0.45	-	-
• Moderate-Light			-	-	2.11(0.85-5.26)	0.11	-	-	-	-	-	-	-	-	-	-	-	-	-	-
Family Type<br>Nuclear- Joint	1.29(0.71-2.34)	0.40	1.07(0.60-1.94)	0.80	1.10(0.60-1.98)	0.76	0.79(0.44-1.44)	0.45	0.83(0.46-1.50)	0.53	-	-	-	-	0.98(0.54-1.76)	0.94	0.87(0.48-1.57)	0.65	0.87(0.44-1.69)	0.67
Partner Presence	1.09(0.63-1.86)	0.76	1.26(0.74-2.16)	0.40	1.36(0.79-2.36)	0.27	0.79(0.46-1.35)	0.39	1.59(0.92-2.75)	0.09	1.01(0.59-1.73)	0.96	1.19(0.69-2.05)	0.52	0.68((0.39-1.16)	0.16	0.79(0.46-1.35)	0.39	0.84(0.47-1.52)	0.58
Partner Satisfaction																				
• Satisfied-Unsatisfied	0.38(0.03-4.47)	0.45	-	-	-	-	1.76(0.14-21.47)	0.66	0.64(0.05-7.83)	0.73	0.73(0.06-8.92)	0.81	-	-	0.39(0.04-4.74)	0.46	0.73(0.06-8.92)	0.81	4.40(0.35-54.37)	0.25
• Very satisfied-Unsatisfied	0.46(0.04-5.24)	0.53	-	-	-	-	2.13(0.19-23.93)	0.54	0.51(0.04-5.76)	0.59	0.48(0.04-5.34)	0.55	-	-	0.50(0.04-5.65)	0.58	0.49(0.04-5.44)	0.56	5.40(0.48-60.74)	0.17
Self-reported sleep quality																				
• Good-Average	1.13(0.58-2.20)	0.71	1.22(0.63-2.37)	0.56	1.70(0.87-3.30)	0.12	0.81(0.42-1.57)	0.54	0.71(0.37-1.39)	0.32	1.40(0.72-2.73)	0.32	0.88(0.45-1.71)	0.71	-	-	0.96(0.49-1.85)	0.89	1.23(0.60-2.53)	0.57
• Very Good-Average	1.47(0.61-3.52)	0.38	1.06(0.42-2.55)	0.89	1.50(0.62-3.60)	0.36	1.28(0.53-3.09)	0.58	0.86(0.35-2.07)	0.73	2.00(0.83-4.86)	0.12	1.67(0.67-4.12)	0.27	1.91 (0.79-4.65)	0.15	1.12(0.47-2.69)	0.79	1.07(0.41-2.75)	0.89

AE-Appearance Evaluation, *AO*-Appearance Orientation, *HE*-Health Evaluation, *HO*-Health Orientation, *FE*-Fitness Evaluation, *FO*-Fitness Orientation, *IO*-Illness Orientation, *BASS*-Body Areas Satisfaction Scale, *OP*-Overweight Preoccupation, *ScW*-Self-classified Weight, *BMI*- Body Mass Index, *OR*- Odd's Ratio, *CI*- Confidence Interval
